# Special Issue “Enterococci for Probiotic Use: Safety and Risk”: Editorial

**DOI:** 10.3390/microorganisms10030604

**Published:** 2022-03-12

**Authors:** Mohamed Zommiti, Sylvie Chevalier, Marc G. J. Feuilloley, Nathalie Connil

**Affiliations:** Research Unit Bacterial Communication and Anti-Infectious Strategies (UR CBSA), University of Rouen Normandie, 27000 Evreux, France; sylvie.chevalier@univ-rouen.fr (S.C.); marc.feuilloley@univ-rouen.fr (M.G.J.F.); nathalie.connil@univ-rouen.fr (N.C.)

Microorganisms, their activity, and metabolites are now considered as intrinsic elements of the human body and this awareness gave was leading to the concept of holobiont [1]. Amongst microorganisms, *Enterococcus* spp. represents a versatile genus. Enterococci, belonging to the group of lactic acid bacteria (LAB), are considered as ubiquitous microorganisms, showing an astonishing potential to inhabit diverse environments, hostile biotopes, and even the gastrointestinal tracts of humans and warm-blooded animals [2]. The main representatives of this genus are *Enterococcus faecalis* and *Enterococcus faecium*. These latter are deemed as the most important strains used for food fermentation and spoilage, but have also been reported as probiotics for more than two subsequent decades without any reports of adverse effects [3]. Under a commercial label, several enterococcal strains have been commercialized as probiotics. For instance, *E. faecalis* Symbioflor 1 (SymbioPharm, Herborn, Germany), which is used to prevent and/or treat diarrhea in pigs, poultry, livestock, and pets, and to treat recurring illness in the human upper respiratory tract. *E. faecium* SF68^®^ (NCIMB 10415; Cerbios-Pharma SA, Barbengo, Switzerland), which is extensively used as a feed supplement for various animals and as a pharmaceutical in humans [4], and *E. faecium* strain 11181, that has been authorized by the European Food Safety Authority (EFSA) Panel as a feed supplement for fattening and enhancing the growth performance of many animals [5].

In terms of global legislation about the use of Enterococci as probiotics, several countries and international legislative agencies have been deeply implicated in this issue. For example, the EU council regulations 700/524/EEC and EG 1831/2003 allow the use of all *Enterococcus* species and strains in poultry, cattle, and pigs. Likewise, in the USA, probiotics used as feed supplements, also known as direct-fed microorganisms, encompass six enterococcal species and are considered as safe; these include *E. faecium* but exclude *E. faecalis*. Of note, *Enterococcus* spp. genus neither has Generally Recognized as Safe (GRAS) status nor has it been included in the Qualified Presumption of Safety (QPS) list implying drastic legislation towards these microorganisms. Recently, the EFSA was bending on developing the QPS system to regulate the use of microbial strains in foods and to ask the probiotic producers’ accountability for assessing the safety for each enterococcus [3].

In the last few years, Enterococci have gained a measure of notoriety, which could not have been reliably predicted a generation ago. Because the fact that they are “tough bugs”, Enterococci can unusually survive for long periods on environmental surfaces and are tolerant to heat, chlorine, some alcohol preparations, and even in antibiotic fields. Such versatility makes of their control a critical task and highly challenging once established in a hospital environment [6]. This is particularly remarkable because Enterococci are not especially virulent microorganisms. 

When we found *Bacillus* spp. with several representatives heading the commercial sector of probiotics, with various probiotic products in different countries all through the globe, here comes out the real question that seeks an urgent answer; why is *Enterococcus* spp. not considered a real probiotic despite its belonging to the LAB group? In terms of sturdiness, *Bacillus* spp., with strains such as *B. anthracis* and *B. cereus*, seem to be more virulent than enterococcal species. For instance, *Bacillus subtilis* causes severe food-borne diseases [7]. At least one *B. subtilis* strain carries all three genes required to produce the Hbl enterotoxin normally produced by *B. cereus* [8]. *Bacillus* spp. are used widely in transformation, whereas the plasmid encodes conjugative or mobile elements [9]. The commercial *B. cereus* IP5832 (Bactisubtil^®^) was isolated from the stool of patients with diarrhea [10]. The strain was later shown to carry genes coding for enterotoxin [11]. Similarly, at a market scale, several probiotic *Bacillus* spp. based products, such as BioGrow^®^ (Provita Eurotech Ltd., Omagh, UK), BioPlus^®^2B (CHR. Hansen, Hoersholm, Denmark, EU approved) and AlCare™, (Alpha-pharmaInc, Melbourne, VIC, Australia, not licensed in the EU) are being used for animal feed and aquaculture [12]. These strains are marketed as antibiotic-resistant probiotics, reflecting a high risk of transferring antibiotic-resistance genes to commensal and pathogens in human and animal gut, and therefore releasing drug resistance genes to the environment through feces [13,14]. 

This Special Issue on “Enterococci for Probiotic Use: Safety and Risk” aims to contribute to the visibility of some of these enterococcal strains as relevant probiotics and contains two research papers and two reviews, presenting the most relevant list of virulence factors associated to enterococcal strains for probiotic use. The research paper by Deng et al. [15] strikingly reported significant evidence for hemolytic activity, label inaccuracy, high level of contamination of *E. faecium*, and the lack of active ingredients in probiotic products for human, animal, aquaculture, and plant use, with the presence of high virulence determinants in probiotic *E. faecalis* and *E. faecium* and other contaminated enterococci, irrespective of their origin. However, the study performed by Scardaci et al. [16] tried to assess the effects of norepinephrine and serotonin treatments on *E. faecium* NCIMB10415 interaction with the human host. Within this context, the authors found that the application of both neuroactive molecules can stimulate the probiotic potential of *E. faecium* NCIMB10415. Regarding detected modifications, the presence of a putative sensor for these molecules has been suggested in this probiotic and was evaluated using *in silico* analyses and micro-scale thermophoresis (MST) technology. 

As the subject of the first review article, led by Krawczyk and collaborators [17] and published in this Special Issue, both the beneficial properties of the Enterococci and the risk factors related to their evolution towards pathogenicity are reported. In this same line, Ferchichi et al. [2] shed light on the world of hurdles and limitations that hamper the *Enterococcus* spp. genus and its representatives from being used, or proposed for use as, probiotics. The future of enterococci use as probiotics and legislation in this field are also discussed.

It is pertinent to mention that the remarkable progress outlined above endorses the urgent call for new recommendations in terms of probiotic regulation and legislative frameworks in order to discern between safe and potentially harmful Enterococcal strains. Leading organizations in food safety and security, such as the European Food Safety Authority (EFSA), the Advisory Committee on Novel Foods and Processes (ACNFP), and the Food Standards Agency (FSA), have allowed the use of certain strains of enterococci as a food additive and supplements based on a careful case-by-case appraisal. In this case, every single strain must be considered, and health risks must be excluded for this specific strain.

Last but not least, in the probiotic realm, it seems better to open the gate to a friend which is too far to be a foe. With an array of proven benefactions during centuries, *Enterococcus* spp. and its representatives seem to be the probiotics of tomorrow, if not in the near future, certainly in next few decades. Till then, accuracy of experimental trials and case-by-case assessments encompassing whole-genome sequencing and deep-learning analyses seem to be the most relevant tools for enterococci to be used as probiotics.

## Data Availability

Not applicable.
